# Mapping provider and consumer voices using the AACTT framework: a focus group study of advance care planning

**DOI:** 10.1186/s12913-025-12240-8

**Published:** 2025-01-21

**Authors:** Lisa Guccione, Stephanie Best, Sonia Fullerton, Sanchia Aranda, Jill J. Francis

**Affiliations:** 1 Department of Health Services Research, Peter MacCallum Cancer Centre, Melbourne, Australia; 2 Sir Peter MacCallum Department of Oncology, Faculty of Medicine, Dentistry and Health Science, The University of Melbourne, Melbourne, Australia; 3 Department of Oncology, Peter MacCallum Cancer Centre, Melbourne, Australia; 4 School of Health Sciences, The University of Melbourne, Melbourne, Australia; 5 Victorian Comprehensive Cancer Centre Alliance, Melbourne, Australia; 6 Centre for Implementation Research, Ottawa Hospital Research Institute, Ottawa, Canada

**Keywords:** AACTT Framework, Process mapping, Complex interventions, Methods

## Abstract

**Background:**

The provision of healthcare is complex. When evidence-practice gaps are identified, interventions to improve practice across multi-level systems are required. These interventions often consist of multiple interacting components and behaviours. To effectively address these complexities, it is crucial to first identify the specific roles and actions required at each stage of the intervention. This approach enables a thorough examination of what is working well and what needs to be optimised. The *action*, *actor*, *context*, *target, time* (AACTT) framework provides a consistent approach to identifying key elements such as ‘who’ (actor) does ‘what’ (action), ‘where’ (context), ‘to or with whom’ (target) and ‘when’ (time). To our knowledge the AACTT has not yet been applied: 1) to specify complex interventions across patient journeys; and 2) to investigate consumer views, despite the importance of patient-centred care.

**Aim:**

Using advance care planning (ACP) as an exemplar complex healthcare process, we describe a method for using the AACTT framework to 1) map a complex model of care across a patient journey 2) capture the consumer perspective; and 3) operationalise these perspectives by comparing across groups and identifying alignments or misalignments.

**Methods:**

Two groups were recruited (healthcare professionals and consumers). Informed by the AACTT framework, four focus groups discussed the process of ACP across existing care pathways. Maps visually representing the perspectives and preferences of healthcare professionals and consumers were co-created iteratively. Qualitative data was deductively coded to the AACTT framework and inductively coded to identify themes within domains. Maps were circulated for critical feedback and refined.

**Results:**

Healthcare professional (n-13) and consumer perspectives (*n* = 11) highlighted what is ‘currently occurring’ in practice, what is ‘not occurring’, and what ‘should be occurring’ to align practice with consumer preferences of care. Comparing participant perspectives identified that most misalignment occurred within the *actor*, *context*, and *time* domains. Misalignment was found predominantly in *actions* ‘occurring sometimes’, with no converging perspectives reported for the *context* and *time* domains.

**Conclusion:**

This novel application of the AACTT framework systematically brings in the consumer voice in ways that may influence the delivery of care. This approach to specifying healthcare professional and consumer perspectives across a complex care pathway identifies barriers that are not found with traditional mapping methods or in current applications of the AACTT framework.

**Supplementary Information:**

The online version contains supplementary material available at 10.1186/s12913-025-12240-8.

## Contributions to the literature

- This paper describes and exemplifies a method for applying the AACTT framework in a novel way to map the provider and consumer perspective and preferences across a complex healthcare process.
- Comparing the alignment and misalignment of perspective and preferences of healthcare providers and consumers can inform where and how to direct change with the aim of potentially improving uptake of healthcare interventions and patient outcomes.
- Using this approach and the AACTT framework to specify the healthcare professional and consumer perspective across a complex care pathway identifies barriers that may impact the implementation and uptake of a healthcare intervention which cannot be identified with traditional mapping methods or identified in the current applications of the AACTT framework.

## Background

The provision of healthcare is complex, often requiring a multi-level systems approach. This complexity is evident in the number of healthcare interventions described as either ‘multidisciplinary models of care’ [1–3], ‘care pathways’ [4–6], or processes to enable ‘continuity of care’ [7, 8]. When evidence-practice gaps are identified, implementation interventions aim to improve practice across multi-level systems. These multifaceted interventions involve various processes and multiple stakeholders, across several steps in patient journeys. Such implementation interventions are also defined as complex interventions consisting of several interacting components with a number of behaviours, targets, and outcomes, where effective delivery requires advanced competencies [9].

Several implementation frameworks propose that before designing implementation strategies, specifying the target behaviours is an essential first step [10–12]. Specifying complex processes and pathways of care is important for assessing fidelity, identifying where evidence-practice gaps occur, and informing the design of interventions to close these gaps. Process mapping is a method used in healthcare settings, as part of quality improvement initiatives, to improve processes of care and understand their complexity within local contexts [13]. It provides a structured picture of complex processes identifying a step-by-step flow that can be measured and studied [13, 14]. Process mapping is presented from the perspective of healthcare providers who currently perform the process [15]. In contrast, an emerging concept referred to as ‘journey mapping’ visually presents touchpoints across healthcare processes but from the patient perspective [16, 17]. This method is like process mapping, but reflects the patient perspective [16]. Journey mapping considers the emotional experiences and preferences of healthcare consumers across the care trajectory. The mapping process not only identifies the current gaps between desired and actual behaviours but also provides actionable insights into how these behaviours can be aligned and, can be used to improve processes by identifying patient preferences and satisfaction at different touchpoints across a care continuum. Specificity is key in both approaches; however guidance on the level of specificity in creating these maps is limited in the peer reviewed literature [13, 17].

It has been proposed that specifying ‘who needs to do what differently’ can help to make the desired processes actionable [18]. The action, actor, context, target, time (AACTT) framework aims to specify single behaviours and sequences of multiple behaviours at different levels of a healthcare organisation, and provides a structured approach to identifying key elements: who needs to do what; where; to, with or for whom; and when [19]. For example, applying the AACTT framework to specify a simple healthcare intervention such as screening stroke patients involves specifying the *actor*, physician; *action*, performing functional outcome measure testing; *context*, rehabilitation centre; *target*, patients with chronic stroke; and *time*, 3–4 months post stroke [20]. A full citation search (October 4th, 2023) of the AACTT framework proposed in Presseau et al. (2019) yielded 143 citations, of which 57 reported empirical studies applying the framework. AACTT was used to inform investigations into barriers and enablers (24 citations), to operationalise clinical recommendations or implementation strategies (16 citations), to code guidelines, policy documents or strategies (13 citations), and to specify behaviour in an interview or questionnaire (4 citations), all of these involving data from the healthcare provider perspective. It thus appears that there are two areas where AACTT has not yet been applied: 1) to specify complex interventions across patient journeys; and 2) to investigate consumer views, despite the growing evidence recognising the importance of patient-centred care [21, 22].

The present paper reports novel applications of the AACTT framework in the context of advance care planning (ACP) within oncology settings to *a)* develop a map that specifies behaviours across the phases of ACP according to the AACTT framework from the healthcare provider and consumer perspectives, and *b)* identify the alignment of the consumer perspective and preferences of care with the healthcare professional perspective. ACP is an appropriate case to demonstrate this because current participation rates are low [25–28] and ACP is a complex journey that requires active engagement of healthcare providers and consumers [29]. The objective of ACP is to enable healthcare providers to take patient preferences and wishes into account, in the event that they are unable to express those preferences at the point of care. It enables individuals to define and discuss goals and preferences for future medical treatment and care with family and healthcare providers, record these goals and preferences, and review them where appropriate [29]. When successful, the benefits of ACP are significant, improving quality of care [30], facilitating timely access to palliative care services [31], reducing families’ and carers’ psychological distress [32], and reducing the cost of care by minimising unwanted aggressive treatments and escalations to acute care [33, 34] and intensive care units [35]. Yet despite practice guidelines and recommendations that ACPs be completed for all people with cancer, ACP is underused, and documentation rates remain low internationally [25–28]. ACP is proposed to occur over three phases: i) preparing for and initiating the ACP conversation; ii) producing the ACP document; and iii) accessing and enacting a documented ACP at the point of care [36, 37]. The AACTT framework was selected due to its focus on specifying multiple behaviours and interactions in a structured manner, particularly useful in complex interventions like ACP. The AACTT framework allows us to address the specific 'who,' 'what,' 'where,' and 'when' and ‘to whom’ of behaviours, which is essential for mapping the behavioural components required across ACP phases. This specificity is critical in guiding further exploration of barriers and enablers; and designing targeted interventions to improve adherence to ACP practices. While traditional mapping tools, such as the Functional Resonance Analysis Method (FRAM), have been successfully used to capture the complexity of healthcare processes, the AACTT framework offers a unique advantage by focusing specifically on behaviour specification across different stakeholders. FRAM, as discussed in studies by O'Hara et al. (2020) and Hedqvist et al. (2023), is adept at identifying interdependencies and vulnerabilities within healthcare systems but does not provide the same level of behavioural detail as AACTT [23, 24]. The use of AACTT in this study therefore offers a granular view of the behaviours that drive process effectiveness in ACP. This paper describes and exemplifies a method for applying the AACTT framework in a novel way. Drawing from process mapping and journey mapping, we describe how we applied the AACTT framework to enable a comparison between healthcare provider perspectives and the lived experiences of consumers.

Specifically, we describe how we used the AACTT framework to:

1. map a complex model of care across a patient journey,
2. capture the consumer perspective; and
3. operationalise these perspectives and preferences by comparing these across groups and identifying where alignments and misalignments occur across ACP phases.

## Methods

### Study design and setting

This study was the first phase of a three-phase implementation science project aimed at improving the uptake of ACP within the Peter MacCallum Cancer Centre (PMCC), a world leading comprehensive cancer hospital in Australia. In phase one we sought to understand the process of ACP within this context and to identify opportunities to improve uptake across ACP phases and throughout care pathways. We used a cross-sectional deductive qualitative study design informed by the AACTT framework to create maps of ACP. The Standards for Reporting Qualitative Research (SRQR) reporting guidelines were used for adequate design and reporting of the study [38]. Ethics approval was obtained for this study from the Human Research Ethics Committee of Peter MacCallum Cancer Centre (HREC/88849/PMCC).

### Using the AACTT framework to map the process of ACP

We adopted the conceptual framework criteria for process mapping proposed in Antonacci et al. [13] and modified this to apply the AACTT framework across five steps. The five steps consisted of: 1) preparation, planning and process identification; 2) data and information gathering; 3) map generation; 4) process analysis; and 5) taking it forward (Table 1).

**Table 1:** Modifying the Conceptual framework criteria for developing a process map proposed by Antonacci et al. (2021) applying the AACTT framework

Criteria^a^	Process^a^	Application of AACTT
Step 1: Preparation, planning and process identification	• A service family and the patient/service user groups is clearly identified	• Identify phases of ACP and broadly specified *actions* • Identify key stakeholders (*actor*s and *targets*) across *actions*
Step 2: Data and information gathering	• Information is gathered to inform the process mapping exercise	• Develop focus group material presenting the broadly specified *actions* for targeted discussion
Step 3: Map generation	• Different perspectives from multiple stakeholders are gathered	• Conduct focus groups with multiple stakeholders from different perspectives, *actors* and *targets* • Identify *action/s, actor/s, context/s, target/s*, and *time* across ACP • Generate visual representations of the ACP process with specificity across AACTT domains
Step 4: Process analysis	• Process maps are analysed and additional information gathered during the process mapping exercise, is represented on the final map. The final map is validated by key stakeholders/experts	• Analyse qualitative data, deductively coding to AACTT domains • Seek critical feedback from stakeholders • Iteratively refine the maps as required • Identify converging and diverging perspectives across AACTT domains
Step 5: Take it forward	• Further actions based on knowledge gained from the process mapping are undertaken demonstrating the actual implementation or testing of improvement ideas	• Explore barriers for actions where perspectives and preferences for care are diverging between *actors* and *targets* • Design implementation strategies to improve uptake and align preferences

^a^Taken from Antonacci et al., 2021

#### Step 1: Preparation, planning and process identification

Informed by the AACTT framework and published literature, conversations with healthcare professionals and consumers were conducted to identify key stakeholders important for influencing the implementation of ACP. Individuals identified as *actors* or *targets* across discrete observable actions were recruited to participate in the mapping exercise. *Actors* were defined as those stakeholders who were involved in performing a discrete observable *action* which formed part of ACP. *Targets* were stakeholders for or with whom ACP was performed.

### Participants and recruitment

Two groups were recruited: 1) healthcare professionals and 2) consumers. The aim was to recruit a minimum of 10 and maximum of 15 participants per group to ensure a minimum of 5 participants for the healthcare professionals and for the consumer group focus groups as per recommended sample sizes [39]. Recruitment of the healthcare professionals group involved the study team identifying healthcare professionals representing key disciplines and services currently involved in ACP at PMCC, including medical, nursing, allied health and hospital administrative staff with a range of experience in ACP (including ACP conversations, documentation, recording/retrieval of documentation etc.). A snowballing sampling strategy was adopted via project lead networks and study participants. LG provided potential participants with a participant information sheet and clarified their understanding and willingness to participate.

The consumer group was recruited by inviting current members of the PMCC consumer advisory group and volunteers with lived experience of cancer and varying levels of familiarity with ACP. A summary of the study outlining the purpose and the contact details of the research team was distributed via the consumer liaison at PMCC to the consumer advisory group and current volunteers. Interested consumers were invited to contact the research team. A member of the research team (LG) provided details of the study and answered any questions. Purposive sampling was used to maximise diversity of participants in terms of gender and experience with ACP which can range from those who have had a conversation on ACP but not created a formal ACP document or engaged with materials to prepare for an ACP conversation but not yet had one.

### Eligibility criteria

Participants were eligible if they met the following inclusion criteria:

#### Healthcare professional group

- Cross-sector healthcare professionals involved in ACP including medical, nursing, allied health and hospital administrative staff.
- Working at PMCC.

#### Consumer group

- 18 years of age or above
- Have or had a diagnosis of cancer.
- Are or have been a family member/carer of a person with a diagnosis of cancer.
- Are able to understand English.
- Do not demonstrate psychological or cognitive difficulties that would preclude study participation.

#### Step 2: Data and information gathering

Focus group stimulus materials and an interview guide to facilitate the focus groups were created for each group and used to facilitate targeted discussions [40] on each phase of the ACP process and to lead the focus groups through participant introductions, setting out the ground rules, clarifying aims and any terminology used, and obtaining verbal consent (Supplementary files 1 and 2). Resources such as practice guidelines, protocols, standard operating procedures, and published literature were searched to identify the Australian National Framework for ACP [36] which was used to present a broad scope of ACP. A pilot focus group was undertaken with research and clinical leads to refine the flow and content of the materials.

### Focus groups procedure

Four focus groups were facilitated by two members of the research team (LG, SF). These were held online through Zoom virtual meeting platform and were recorded. The decision to conduct focus groups via video conferencing was made in light of the restrictions posed by the COVID-19 pandemic and the preferences of consumers whom to which some were receiving cancer treatment and wanted to minimise their exposure. Utilising online platforms has advantages and, in this study, enabled participants to engage in discussion and facilitated real-time sharing and iteration of the process maps. We do however also acknowledge the limitations of this method and that this may have limited our recruitment to those with the technological expertise to navigate the platform. Verbal informed consent was obtained from all participants at the start of the focus groups, and facilitators addressed any outstanding issues or questions raised by participants. All participants were reminded that participation was voluntary and that they were able to withdraw from the study at any time without giving a reason.

Participants were asked questions to facilitate discussion on the process of ACP across existing care pathways from registration through treatment and follow-up. An episodic narrative approach [41] was used to track the discussion of experiences across care pathways relative to the phases of ACP and to develop maps. The AACTT framework was used to guide behaviour specification, as described below. The Australian National Framework of ACP [36] was presented as a starting point to focus discussion and map behaviours across the phases of ACP.

#### Step 3: Map generation

The focus group stimuli were presented defining: 1) the evidence-practice gap; 2) terminology; and 3) the key actions for ACP proposed in the Australian National Framework consisting of i) having the conversation, ii) making the document, and iii) accessing and enacting the ACP document using a schematic diagram. This figure was used as a starting point to iteratively create the maps, with participants building on the figure by firstly exploring the *actions* presented in the schematic and any additional *actions* that form part of the complex intervention that were not presented; identifying the *actors* across each of the actions, discussing the *context* in which these actions occur; identifying the *target* for whom these actions are performed for; and the *time* when the actions occur. Maps were created interactively with participants. This resulted in mapping of the process from two perspectives, one from the healthcare provider perspective and one from the consumer perspective.

#### Step 4: Process analysis

Qualitative data from video recorded focus groups were transcribed and deductively coded to the AACTT framework and inductively coded to identify themes within the AACTT domains. For example, there were *actions* that 'occurred', 'sometimes occurred', or 'did not occur' but 'should occur'. Similarly, there were actors who 'could perform the action', who 'should perform the action', and who 'do perform the action'. Maps presenting the actors’ perspectives and the targets’ perspectives were then merged to identify alignment across the complex intervention and opportunities to identify potential points to target for improvement.

Maps were developed and circulated by email to participants for further comments and feedback within a week of the focus group meetings. All feedback was incorporated within the maps and circulated to participants for a second time. To ensure data protection, participant feedback on the maps was collected via secure institutional email systems. All participants provided informed consent, and the methods used were consistent with institutional data security guidelines. Although the primary design of this study was qualitative, quantitative comparisons were made to better illustrate the misalignments between healthcare professional and consumer perspectives. Numerical summaries were provided to indicate how frequently certain behaviours (e.g., having an ACP conversation) were reported as 'occurring sometimes' or 'not occurring.' These comparisons were made using thematic analysis to categorise behaviours, followed by a frequency count to quantify the prevalence of each behaviour across focus group discussions. This methodological approach within qualitative research is used to emphasise the importance, frequency, or strength of findings [42, 43]

#### Step 5: Taking it forward

Opportunities to improve participation with ACP were identified to inform recommendations for the next phase of the project.

## Results

Four focus groups were conducted with 24 participants. Representativeness across groups in terms of discipline for the healthcare professionals, and ‘experience with ACP’ for consumers, was obtained below the maximum sample size of 15, see Table 2. For pragmatic reasons we were unable to achieve equal distributions across genders in our groups.

**Table 2:** Demographics of participants in the healthcare professionals and consumers groups

**Focus Groups**	**Number of participants**
***Healthcare professionals***	
Disciplines	
- Medical (haematologist, radiation oncologist, palliative care physicians)	4
- Nursing	4
- Allied Health	2
- Hospital administration	3
Gender	
- Female	12
- Male	1
Total—Healthcare professional participants	**13**
***Consumers***	
Participants with an ACP document	
- Has an ACP	4
- Does not have an ACP	6
Has had an ACP conversation	
- Yes	9
- No	2
Language spoken at home	
- English	11
Languages other than English	0
Gender	
- Female	9
- Male	2
Total—Consumer participants	**11**

### Using AACTT to map ACP

#### Specifying ‘actions’ across the phases of ACP

**Fig. 1:**
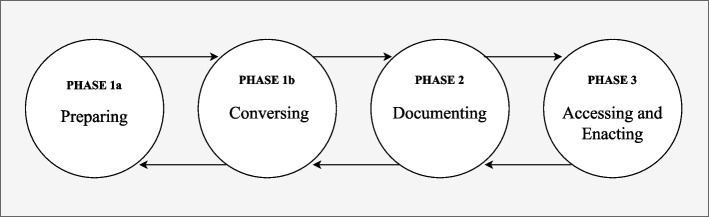
Adapted from the phases of ACP proposed in Guccione et al. (2023)

The three phases proposed in the Australian National Framework of ACP [36] were discussed in the context of the AACTT framework. Healthcare professional and consumer focus groups identified various actions associated with each of the phases of ACP. Clusters of actions aligned with the preparatory phase 1a proposed in Guccione et al. ^21^ and were mapped as a phase of ACP (Fig. 1). Focus group participants discussed their experiences on who could do what, where, to whom, and when at the point of care across each of the phases.

*Actions* were discussed in three themes: i. regularly occurring, ii. sometimes occurring, and iii. not occurring but should be, from healthcare professionals’ perspectives or ‘would be helpful’ from consumers’ perspectives. Actions coded as ‘sometimes occurring’ were those reported inconsistently by participants and a theme failed to be identified.

### Actions regularly occurring

Healthcare professionals identified 12 actions that were regularly occurring across the phases of ACP with most of these associated with Phase 1a. Healthcare professionals reported more actions as regularly occurring (75%) than consumers (28%) across the phases of ACP. At phase 1a, only one of these actions reported by healthcare professionals (providing written information and resources on ACP) aligned with what was reported from consumers (receiving written information on ACP). The action of 'explaining ACP' and 'having ACP explained' aligned across health professional and consumer perspectives but was reported by consumers to occur only sometimes.

Three actions were identified in Phase 1b, which aligned from the perspectives of both groups. Healthcare professionals reported all three actions were occurring. Consumer perspectives aligned with two actions but reported that ‘having the conversation about goals of care’ occurred only sometimes. Actions were not aligned at Phase 2 of ACP. Healthcare professions identified three actions associated with 'making an ACP document', yet consumers reported that' creating the ACP document' only occurred sometimes. Perspectives of healthcare professionals and consumers aligned in phase 3 with both groups reporting that in the absence of an ACP, healthcare professionals referred to Phase 1b with family or a substitute decision maker.

### Actions occurring sometimes and actions not occurring

From a total of 16 actions identified by healthcare professionals, 25% were reported as sometimes or not occurring (2 actions occurring sometimes and 2 actions not occurring) compared to 71% identified from a total of 14 actions by consumers (5 actions occurring sometimes and 5 action not occurring). At phase 1a, three actions were identified as not occurring. These were not discussed by healthcare professions but identified by consumers as actions in the preparation for the ACP conversation that would be helpful.

Healthcare professionals and consumers discussed actions that were necessary for Phase 3 but did not currently form part of the ACP framework. This phase has been proposed in this paper as phase 2b and consisted of two actions reported by healthcare professionals and consumers as actions that are not occurring but should be. Actions in phase 3 'enacting and accessing an ACP' were reported by healthcare professionals and consumers as sometimes occurring. Those that described their experiences of an ACP not being accessed or enacted related this to actions associated with phase 2b, as ACP had not been successfully followed-up or uploaded to the system to which it could be accessed.

### Actor/s, Context, Target, and Time for Actions occurring sometimes and actions not occurring

Deductive analysis identified themes across the phases of ACP for actions that were occurring sometimes, and actions that were not occurring but should be or would be helpful depending on the perspectives and preferences expressed by healthcare professional and consumer groups. These are presented in Table 3.

**Table 3:** Comparison of themes across AACTT domains for actions occurring sometimes and not occurring

Action/s across phases of ACP	Actor/s	Context	Target	Time
**Phase 1a**				
***Explaining ACP/ Having ACP explained***				
HPC perspective: Having *“preparative conversations”, “commencing that ACP conversation to explain what it is”*	Allied health, nurses, doctors	“*At admission screening*”, in *“outpatient, inpatient settings”*	With the patient	*“Often requires several clinic appointments”*
Consumer perspective and preferences (ACP verbally explained also want the role of a substitute decision maker explained) *“Who that person is”, “miscommunication about that role”*	Preference for clinical nurse, allied health, lawyer *“more comfortable if their lawyer explains it to them ….It just made us feel better, I think”*	Want ACP explained ‘face to face’ in the context of making a will rather than GOC *“a massive part that’s missing is a face-to-face interaction”*	With the patient	Sometimes occurring. Preference is when receiving written information and resources
***Providing/receiving written information and resources on ACP***				
HPC perspective: Provide/receive *“written information and resources on ACP”*	Health admin, doctors, nurses	“*Provided at registrations*”, *“outpatient, and inpatient settings”*	To “*new patients*” and those that are “*completely unfamiliar”* or “*would like receive further information”*	*“When registering a patient, anytime, if deteriorating, or treatment stopping”*
Consumer perspective and preferences (want information on how to approach discussions with family) *“some dot points to take home to discuss with significant others”*	Health admin	With other materials about Peter MacCallum Cancer Centre	To the patient	During registration; least important information more relevant after treatment
***Scheduling an appointment for future ACP discussion***				
Not raised by HCPs	-	-	-	-
Consumer perspective and preferences (this does not occur, consumers preference reported) *“you have an appointment set up for you with a such and such, and you'll sit down and start that process”*	**?**	At admission, in the context of routine practice *“then it's not attached to that you're gonna die. It's just another part of the sequence of events”*	With the patient	Not occurring. Preference at admission before conversations about GOC *“at the beginning of my admission into Peter Mac was the right time”*
**Phase 1b**				
***Having a conversation about GOC***				
HPC perspective: GOC conversation around the preferences of care	Nurses, allied health doctors “*medical team*”	At *“nursing admission point” and “in the context of where their illness is at”.* Ideally would have a separate clinic	Most often with patient, some conversations with family	*“Points in care trajectory change”, “when you know the patient Isn't doing well, and things are not gonna go well, and you have to start putting limits on care”*
Consumer perspective and preferences: GOC conversation around the preferences of care (did not always occur, consumer preference reported)	More comfortable with nursing, allied health, legal professionals, *“we're all sort of saying the doctors are probably not the people we want”*	Not in the context of a change in health status but rather part of routine practice, *“in the context of how would you like to be cared for”*	With the patient and family member/s or nominated substitute decision maker “*I would want my medical decision maker with me”*	Sometimes occurring. Preference for ongoing conversation as preferences change over time, can occur anytime not because of a change in health status. “*It's not a one-off”,* “*needs to occur repeatedly”*
	Patient	Their preferences and the importance of them	With family and or nominated substitute decision maker	Sometimes occurring. Preference is at the beginning of illness and any point along the way
**Phase 2**				
***Creating the ACP document***				
HPC perspective *“signing of the paperwork”, “making the ACP document”*	Doctors, allied health, patient with external professionals	Inpatient and outpatient settings	For the patient	When points in care trajectory change, deteriorating health status
Consumer perspective and preferences	Patient, legal professionals, medical person	Not in the context of a cancer diagnosis part of creating a last will and testament	For the patient	Sometimes occurring. Preference for reviewing intermittently and updated as values and GOC change
**Phase 2b**				
***Follow-up and uploading of ACP documents prepared externally***				
HPC perspective and preferences (currently no follow-up on ACP documents externally prepared) *“someone's already got an ACP and then getting a copy of that. It's been one important concept that we must forget…”*	Should be an administrative role	Hospital registrations	External agencies, GPs, nursing homes, for the patient or with the patient	Not currently occurring. Preference is to follow up at discharge, in clinics or when in the community
HPCs reported a preference for a function for patients to upload ACP documents)	Patient	Online patient portal	HPCs to access	Not currently occurring. Preference when ACPs are completed externally
Consumer perspective and preferences (currently no follow-up of existing ACPs) *“until they follow me up I'm not going to give it to them”*	Health admin	Hospital registrations	With the patient	Not currently occurring at any point
(currently no uploading of ACP documents provided, consumer preference for patients to be able to do this) *“I handed it over: and it never got uploaded into the system”*	?	Patient medical record	HPCs to access	Not occurring *“just assumed it was running..he was not for resuscitation.. not knowing that you had to do it every admission”*
**Phase 3**				
***Accessing and enacting ACP***				
HCPs perspective (not always accessible and therefore not always enacted) *“it's too late in this stage to get to complete the documents”*	Doctors	When the patient can no longer make decisions for their care	For the patient	Sometimes occurring
Consumer perspective (not always accessible and therefore not always enacted) *“his wishes weren't respected”*	Doctors	When the patient can no longer make decisions for their care	For the patient	Sometimes occurring

*Abbreviations: HCP* Healthcare professionals, *ACP* Advance care planning, *GOC* Goals of care

Differences in the perspectives and preferences of healthcare providers compared with consumers across the *actor/s, context, and time*, domains were identified for actions explaining ACP (phase 1a), having the conversation about goals of care (phase 1b), and creating the ACP document (phase 2).

The perspectives and preferences of healthcare professionals differed most from the preferences of consumers on the *who*, *where* and *when* across phases 1a, 1b, and 2. Healthcare professionals specified doctor as an *actor* for explaining ACP, having the conversation about goals of care, and making the ACP document. Consumers consistently reported preferences for not having doctors perform these actions. The context and timing of these actions also differed between stakeholders. Healthcare professionals specified the most appropriate time for these conversations was when there was a deterioration or when points in the care trajectory had changed. Consumers specified that the timing of these actions were most appropriate when not linked with a change in health status and that these actions were in the context of routine care rather than linked with their illness.

Healthcare professional and consumer preferences converged at phase 2b across AACTT domains. Both groups reported following-up and uploading of ACP documents prepared externally to the hospital was an action that was not occurring but a necessary step to improve outcomes associated with Phase 3, accessing, and enacting an ACP document.

### Opportunities to improve ACP

Opportunities were identified to improve ACP across ACP phases 1a, 1b, 2, and 2b. These behaviours were identified as opportunities for improving ACP because they were either reported as not occurring but should be occurring by either the healthcare professional or consumer groups; or reported as sometimes occurring with diverging perspectives across the AACTT domains therefore an opportunity to improve ACP by aligning preferences is noted.

Opportunities to align healthcare professional and consumer perspecitves and preferences occurred at the *actor*, *context*, and *time* domains. Perspectives and preferences of healthcare professionals and consumers differed most across these domains. Aligning preference across these domains are opportunities to improve participation across phase 1a, 1b, and 2.

In summary, the mapping of actions across ACP phases from both healthcare professional and consumer perspectives highlighted what is ‘currently occurring' in practice, what is 'not occurring', and what 'should be occurring' to align practice with consumer preferences of care. In phase 1a, preparing for the conversation, the healthcare professional and consumer perspectives were misaligned in terms of the desired actions. Comparing the perspectives and preferences of healthcare professionals and consumers across AACTT domains identified that most misalignment occurred within the *actor*, *context*, and *time* domains, specifically across phase 1 (preparing for, and initiating the ACP conversation) and phase 2 (creating the document). Misalignment was found predominantly in *actions* that were ‘occurring sometimes’, with no converging perspectives reported for the *context* and *time* domains.

## Discussion

**Fig. 2:**
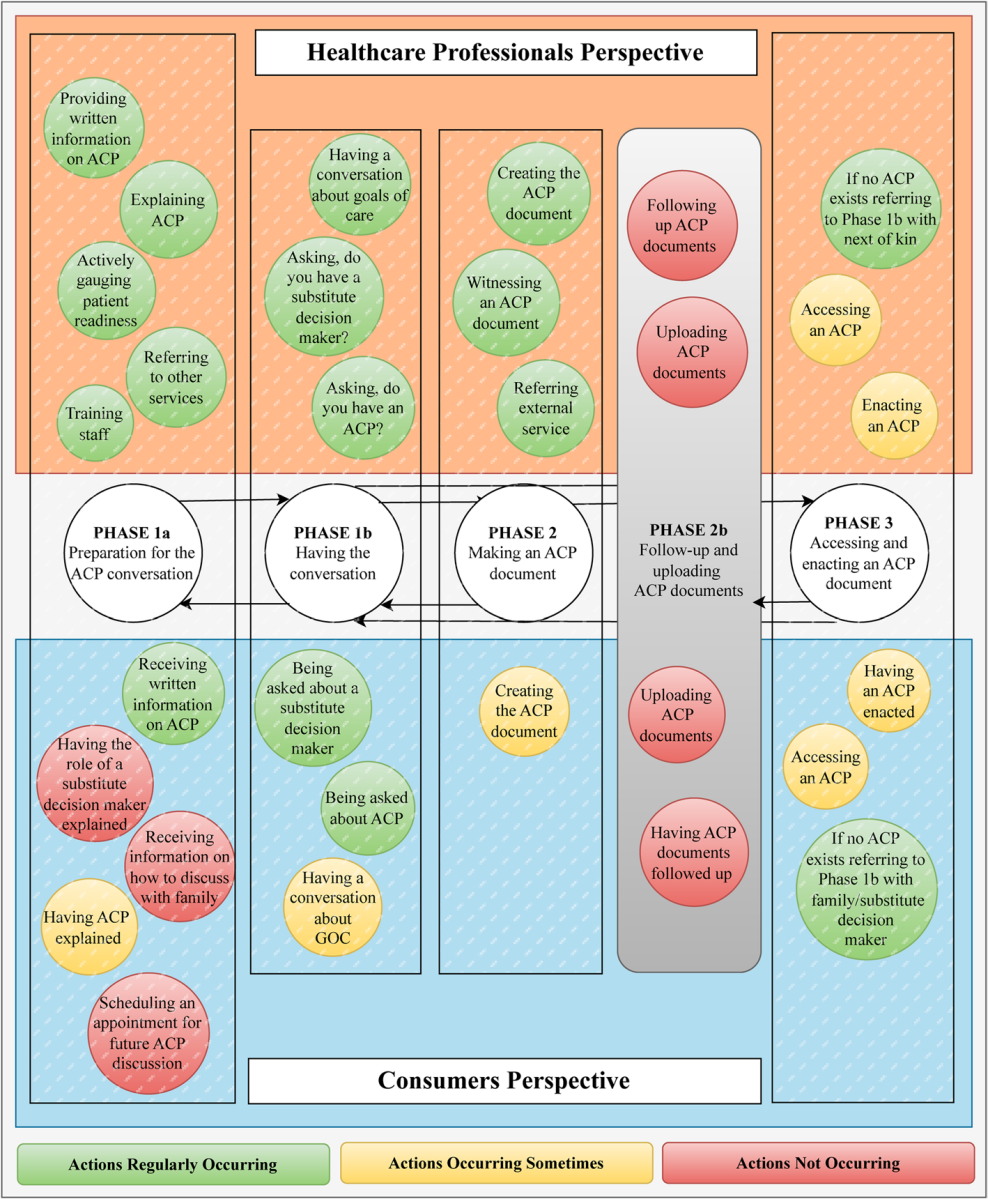
Actions identified across ACP phases from the healthcare professional and consumer groups’ perspectives

**Fig. 3:**
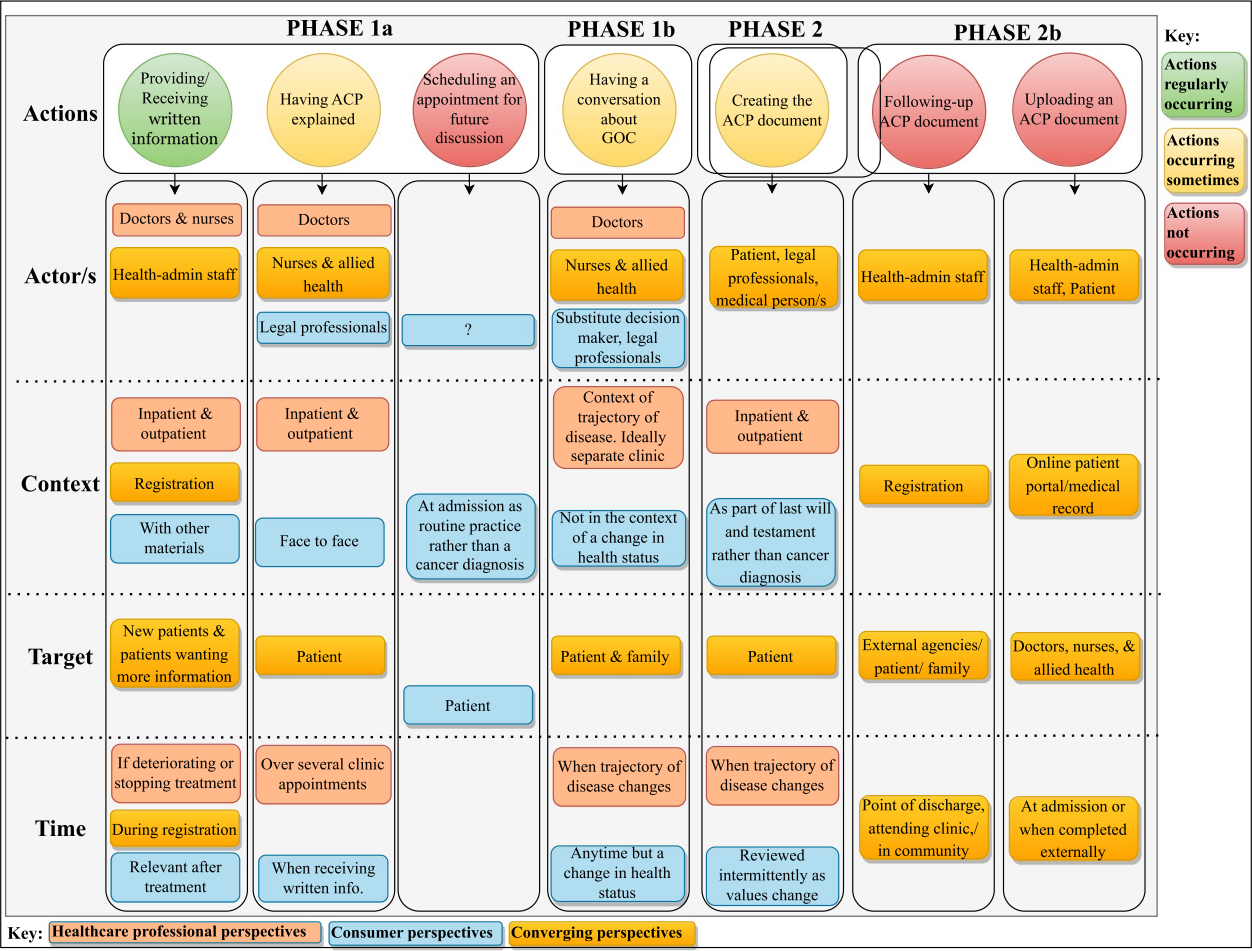
Healthcare professional and consumer perspectives across AACTT for actions sometimes and not occurring

We have illustrated four steps of a five-step method for developing a map of a complex intervention by applying the AACTT framework in a novel way. Combining elements of process mapping [13–15] and journey mapping [16, 17] we have presented the perspectives and preferences of healthcare providers and consumers, mapping a complex model of care (advance care planning), across a patient journey (Fig. 2). By capturing the consumer perspective, we identified alignments and misalignments with the views of healthcare professions in the domains, *actor*, *context*, and *time* (Fig. 3). To our knowledge this is the first time the AACTT framework has been used to inform this methodological approach. We propose that the method that we have described can be applied to a range of complex processes in healthcare settings, especially those where patient engagement might influence the process. While the framework is presented linearly, its application in this study demonstrates how the framework can be applied to account for the dynamic and evolving nature of ACP.

**Fig. 4:**
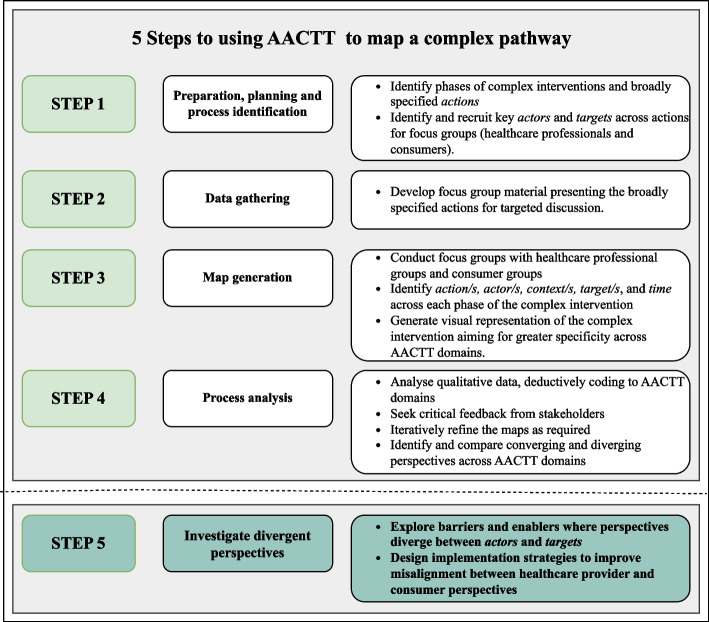
Five-step approach to mapping provider and consumer perspectives using the AACTT framework

Presented in Fig. 4 is a five-step approach that can be used to map the provider and consumer perspectives across a complex model of care using the AACTT framework. We have demonstrated the first four steps in this paper. *‘Taking this forward’*, the fifth step proposed in Antonacci et al. (2021) has been modified in our model. Although not exemplified in this paper, we propose the fifth step as ‘*Investigate divergent perspectives’* and to be consistent with a multilevel approach to process change, propose that this should involve a consultation approach with key stakeholders and prioritisation for investigating misalignment of perspectives. Whilst the consumer voice is important for delivering patient-centred care [21, 22] this may not always be feasible or appropriate in all healthcare contexts. For example, consumers may prefer to have a conversation about their goals of care with legal professionals; however, they may not have the medical knowledge necessary to advise of their options in the context of a cancer diagnosis. Similarly, health policy may dictate the roles and responsibilities of healthcare workers in ways that may not align with patient preference. Therefore, the fifth step of our approach would involve conducting prioritisation exercises to determine which divergent perspectives are appropriate to target with the aim of investigating how care can be delivered in a way that aligns with consumer preferences. Prioritisation can be driven by policy, evidence or prioritisation criteria for example – what is most important for consumers, what is most feasible for healthcare providers, or what is likely to have the most impact on overall uptake. Using the prioritisation process to guide next steps, determinant frameworks such as the Theoretical Domains Framework can then be used to assess barriers and enablers and further inform the development of implementation strategies to improve misalignment between healthcare provider and consumer perspectives.

### Strengths of this method

The main strength of this novel application of the AACTT framework was systematically incorporating the consumer voice with the perspectives of healthcare professionals in mapping a complex healthcare process. This approach highlighted those *actions* important for consumers*,* were not always consistent with those reported by healthcare professionals. Demonstrated in our results the *actions* associated with ‘preparing for having the conversation’. This method allows us to identify gaps in practice. Whilst this can also be done with traditional process mapping [13–15], using the AACTT framework to unify this approach with journey mapping incorporating the consumer voice, gives us an opportunity to align practice with patient preferences. Identifying *actions* that may 'not be occurring' but need to occur to align the delivery of care with patient preferences, will ultimately provide better care for patients and improve outcomes. Whilst incorporating the patient perspective in health research and across many implementation science approaches is best practice, using the AACTT framework to specify behaviours across a complex health intervention from the perspectives of both healthcare professionals and consumers has not been previously explored.

The AACTT framework has unified two mapping approaches to incorporate the views of both healthcare professionals and consumers to enable these perspectives to be operationalised and compared. Evidence suggests that healthcare providers perceptions are not always concordant with the actual beliefs of patients [44, 45]. Using a framework to specify behaviours across a care continuum from both the healthcare provider and patient perspective enables us to compare these perspective and preferences and measure how much and where one deviates from the other. For example – across the phases of ACP percentages of *actions* ‘regularly occurring’, ‘sometimes occurring’, and ‘not occurring’ from a healthcare professional’s perspectives; can be compared with consumer perspectives. Comparing the number of misalignments between groups at specific *actions* can also inform prioritisation for investigating how to align the delivery of care with patient preferences.

Assessing the alignment across AACTT domains adds another layer to this novel methodological approach. Not only can we identify *actions* that are important for a healthcare intervention, but we can also explore the preferences around how this care is delivered. For example—exploring each domain across the AACTT framework for a complex healthcare intervention specifies how a behaviour should be performed. In the context of ACP by incorporating the consumer voice we identified that ‘having a conversation about goals of care’ was not occurring as consistently as what healthcare professionals reported. The perspectives and preferences on how this behaviour occurred also did not align for healthcare professionals and consumers. This approach enabled us to identify at what domain the preferences differed. Healthcare professionals thought that *doctors* were important for *having the conversation about goals of care* and that this should occur *in the context of where they were at in the trajectory of their disease*, with the *patient and family, when there is a change in health status*. The consumer preferences were to have this conversation with *anyone but their doctor, not in the context of their disease and at any time except when there was a change in health status*. This level of specificity can inform how to approach next steps in terms of how to improve uptake of this behaviour and specifically guide step five of this approach, to investigate divergent perspectives. In this case raising questions as to which barriers we consider exploring; those encountered by *actors* (healthcare professionals)? Barriers encountered by *targets* (consumers)? Or do we address barriers preventing the alignment across AACTT domains?

Our approach also identifies barriers that will impact the implementation and uptake of a healthcare intervention that cannot be identified with traditional mapping methods or identified in the current applications of the AACTT framework. We propose that when perspectives and preferences for care are not aligned between healthcare professionals and consumers a ‘meta barrier’ occurs, sitting above domains often used to identify barriers such as those proposed in the theoretical domains framework [11, 46]. This barrier may exist beyond addressing any barriers that prevent healthcare professionals from performing a behaviour. Therefore, implementation strategies designed to target barriers encountered by healthcare professions may not be effective. Even if implementation strategies get healthcare professionals to do something different, changing their behaviour, trying to deliver care in a way that does not align with patient preferences is still likely to result in a disconnect from patients with them unlikely to engage.

### Potential limitations of this method

We acknowledge that this application is not traditional process mapping. Traditional process mapping can identify where inefficiencies lie in completing a process and where system improvements can be made [13–15]. Our mapping does not systematically map in a sequential flow the process of a complex intervention with decision boxes, as does process-mapping. However not all healthcare interventions can be presented in this way, as is the case of ACP. Our approach incorporates the individual lived experience of consumers and creates iterative generalised maps that aim to improve practice by 1) identifying what is occurring, not occurring and what should be occurring and 2) identifying the misalignment and specifying across AACTT domains where this occurs from both consumers and healthcare professionals perspectives so to inform next steps which would involve an exploration of barriers and enablers to align the delivery of evidence based practice with patient preferences.

## Conclusion

This novel application of the AACTT framework systematically brings in the consumer voice in ways that may influence the delivery of care. Comparing the alignment and misalignment of the perspective and preferences of healthcare providers and consumers can inform where and how to direct change with the aim of potentially improving uptake of healthcare interventions and patient outcomes. Using this approach and the AACTT framework to specify the healthcare professional and consumer perspective across a complex care pathway identifies barriers that may impact the implementation and uptake of a healthcare intervention which cannot be identified with traditional mapping methods or identified in the current applications of the AACTT framework.

## Supplementary Information

Supplementary Material 1.

Supplementary Material 2.

## Data Availability

The dataset which consists of focus group transcripts is not publicly available due to confidentiality policies.

## Abbreviations

AACTT: Action, Actor, Context, Target, Time
ACP: Advance Care Planning
PMCC: Peter MacCallum Cancer Centre
SRQR: Standards for Reporting Qualitative Research
HREC: Human Research Ethics Committee
