# Supplementary material for: Mapping provider and consumer voices using the AACTT framework: a focus group study of advance care planning
Source: BMC Health Serv Res. 2025 Jan 21;25:115. doi: 10.1186/s12913-025-12240-8 (PMC11752742; doi:10.1186/s12913-025-12240-8)
Supplement: Supplementary file 1 — Supplementary Material 1. [file 12913_2025_12240_MOESM1_ESM.pdf]

Improving the uptake of advance care planning for people with cancer

Focus Group Stimulus

Peter Mac
Peter MacCallum Cancer Centre
Victoria Australia

OUTLINE FOR THIS SESSION….

- Welcome and brief introductions
- Improving ACP at Peter Mac
- Todays objectives, questions?
- Ground rules and consent
- Discussion
- Next steps

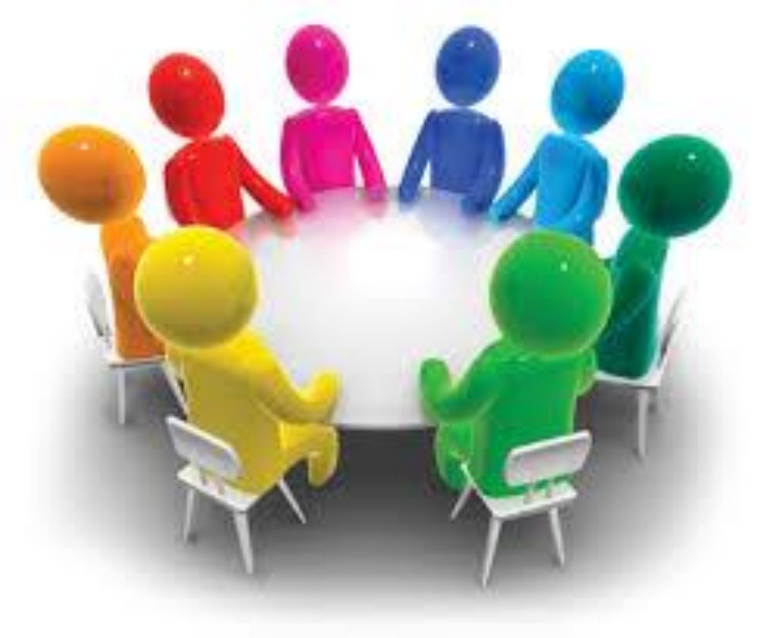

Peter Mac
Peter MacCallum Cancer Centre
Victoria Australia

INTRODUCTIONS

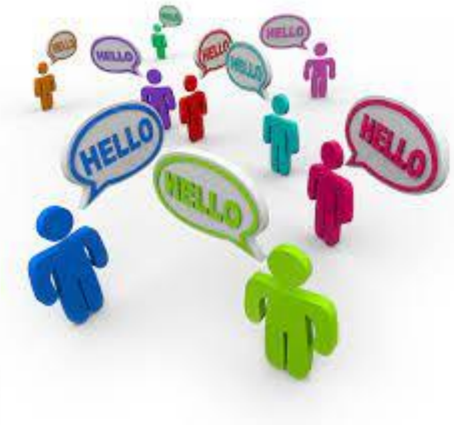

Peter Mac
Peter MacCallum Cancer Centre
Victoria Australia

ADVANCE CARE PLANNING

A process allowing individuals to discuss and record personal values, beliefs and preferences, to guide clinical decision-making in the event they lose capacity to make or communicate their treatment decisions.

“If you were unwell, and could not talk to the doctors and make decisions about your medical treatment….”

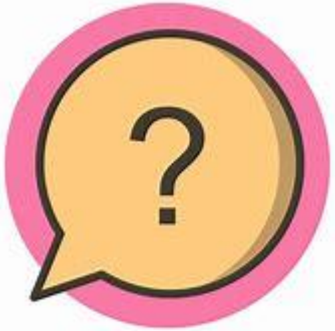

“WHO would speak for you?”

“And WHAT would they say?”

Who? Medical treatment decision maker… appointed vs hierarchy

What? Advance care plan, advance directive (instructional and/or values)

Peter Mac
Peter MacCallum Cancer Centre
Victoria Australia

ADVANCE CARE PLANNING

THE PROBLEM: The DH target for ACP documentation is 50% of inpatients over 75 years.

Peter Mac falls short of this target: 9% of inpatients over 75 years, 6% of all inpatients (epic).

ACP IMPROVEMENT PROJECT

AIM: Working with key stakeholders (patients, clinical staff and support staff, and consumers), co-design care pathway improvements to increase the uptake and documentation of ACP.

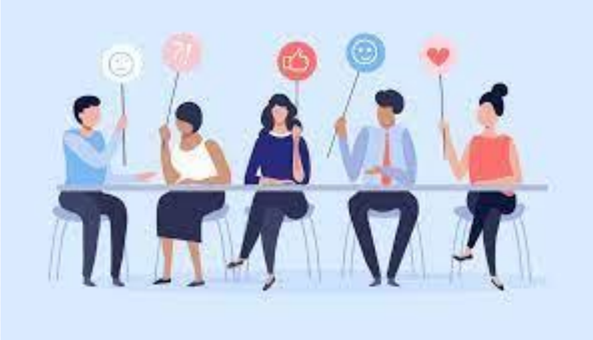

<u>Phase 1:</u> Mapping

Peter Mac
Peter MacCallum Cancer Centre
Victoria Australia

QUESTIONS?

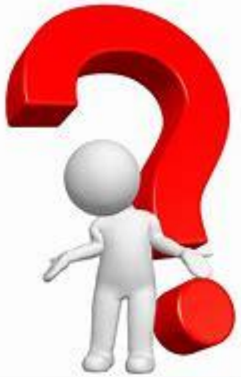

Peter Mac
Peter MacCallum Cancer Centre
Victoria Australia

Ground rules

Do you consent to participate in this study?

- Voluntary participation
- No right or wrong answers, only different points of view. Important to hear about a range of views from everyone here.
- What's discussed during the session is not discussed outside of the focus group session
- We are recording the session – feel free to switch off the video and change the name if you would feel more comfortable
- Any information we use will be de-identified

- My role as the moderator will be to guide the discussion but feel free to talk to each other

Peter Mac
Peter MacCallum Cancer Centre
Victoria Australia

MAPPING ACP AT PMCC

<u>Phase 1:</u> Process map

Focus Group 1: *Clinicians, nurses, allied health, admin*

Focus Group 2: ***Consumers***

Circulate process map: Groups will provide feedback on process map - address any variances.

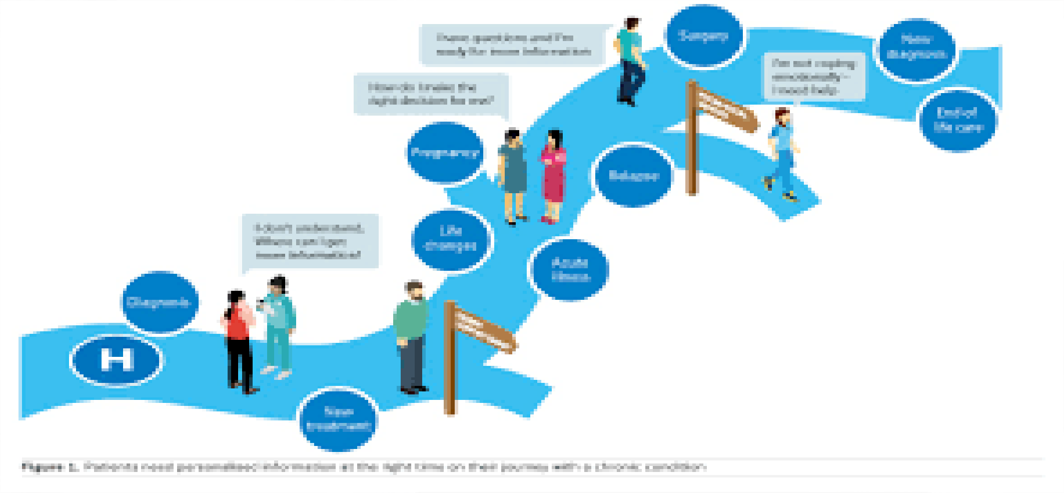

Touchpoints, opportunities, and optimal timing for asking about, discussing, and documenting ACPs for various key staff members and consumers

Peter Mac
Peter MacCallum Cancer Centre
Victoria Australia

ACP – Mapping

Australian National Framework for ACP

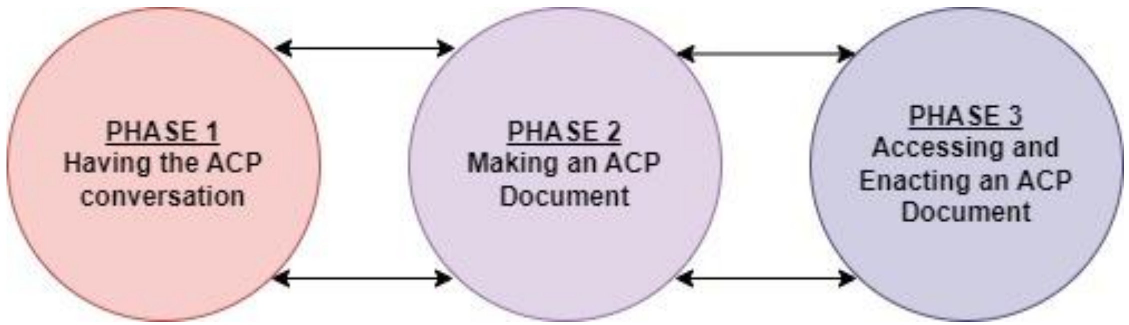

Top circles are a diagram of some of the steps of ACP that have been presented literature. We are interested in exploring

What generally happens across each of these phases? Who are these phases relevant for?

Peter Mac
Peter MacCallum Cancer Centre
Victoria Australia

ACP – Mapping

Australian National Framework for ACP

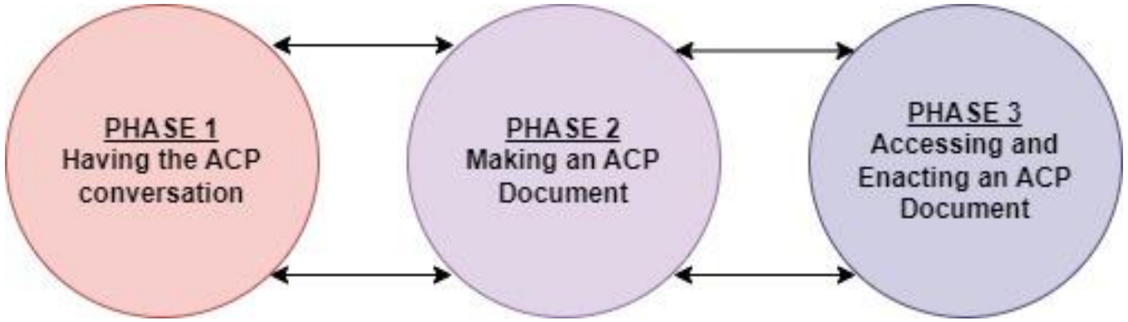

Within your role how are you involved with ACP – across which phases? And in what setting?

Peter Mac
Peter MacCallum Cancer Centre
Victoria Australia

Doctors | Nurses | Allied Health | Administrative | ?

ACP – Mapping

Australian National Framework for ACP

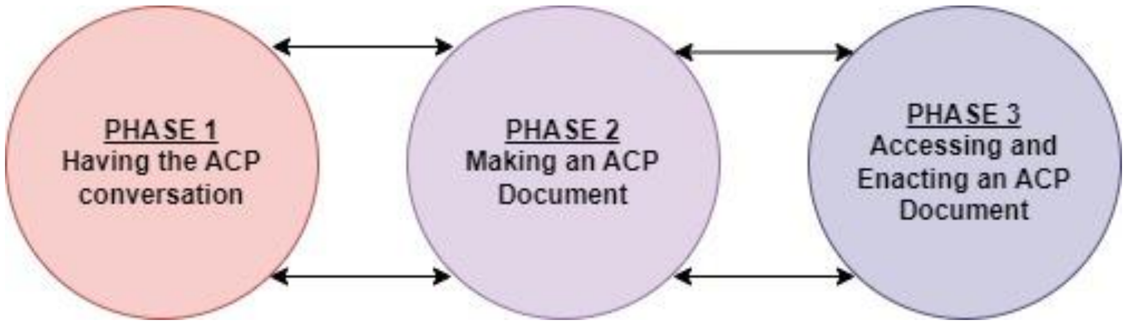

Is there an optimal time?

Peter Mac
Peter MacCallum Cancer Centre
Victoria Australia

PROCESS MAPPING ACP AT PMCC

<u>Phase 1:</u> Process map

Focus Group 1: ***Clinicians, nurses, allied health, admin staff***

Focus Group 2: *Consumers*

Circulate process map: Groups will provide feedback on process map - address any variances.

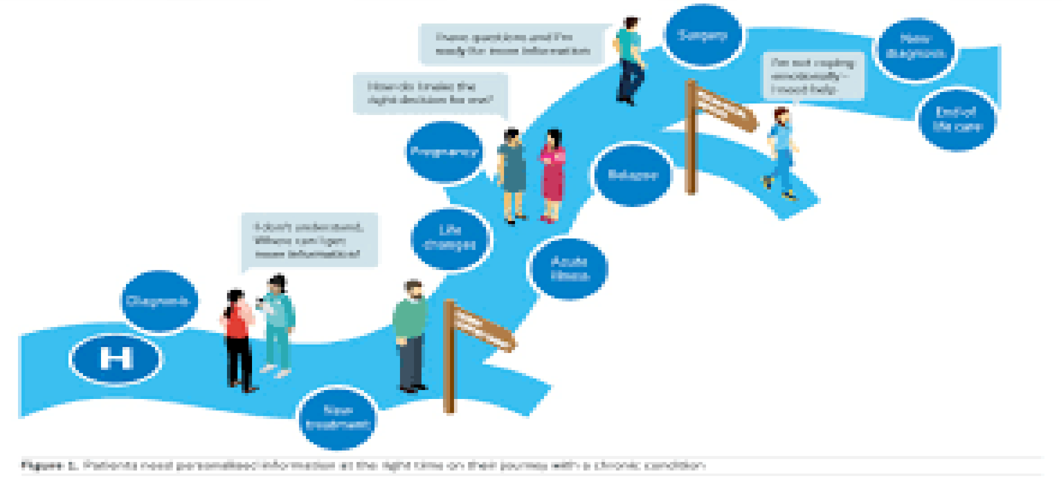

Touchpoints, opportunities, and optimal timing for asking about, discussing, and documenting ACPs for various key staff members and consumers

Peter Mac
Peter MacCallum Cancer Centre
Victoria Australia

ACP – Mapping

Australian National Framework for ACP

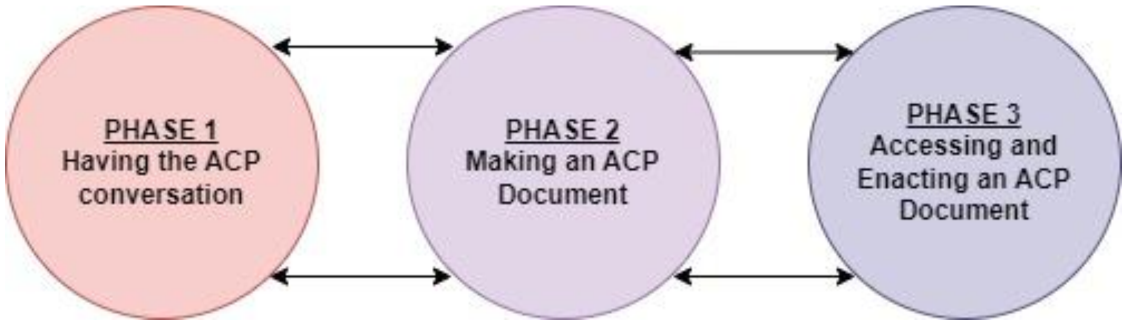

Top circles are a diagram of some of the steps of ACP that have been presented literature.

Thinking of your own experiences what did this process look like for you?

What's missing?

Peter Mac
Peter MacCallum Cancer Centre
Victoria Australia

ACP – Mapping

Australian National Framework for ACP

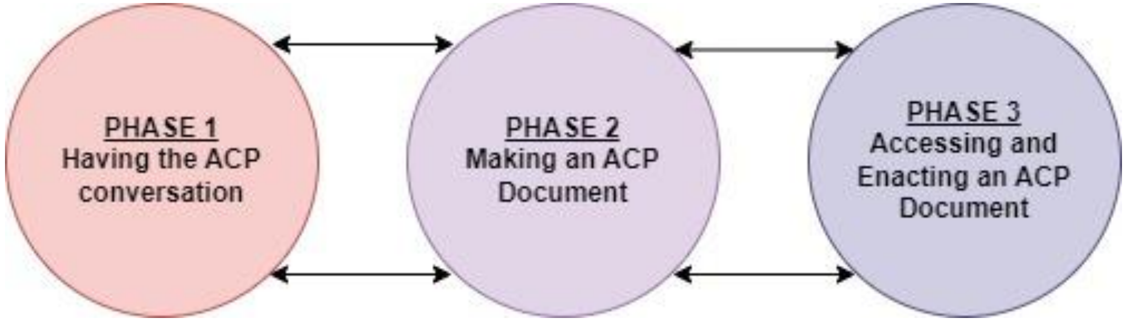

Through your own experiences **who** are the most appropriate people to be involved? And in what setting?

Doctors   Nurses   Allied Health   Administrative   ?

Peter Mac
Peter MacCallum Cancer Centre
Victoria Australia

ACP – Mapping

Australian National Framework for ACP

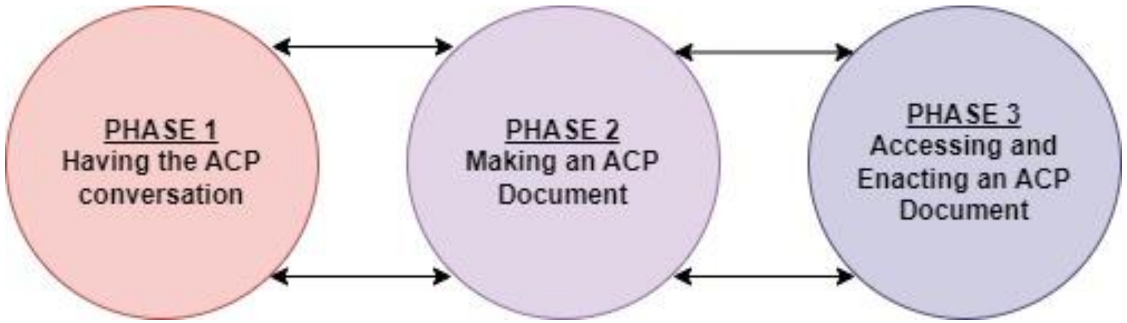

Through your own experiences **when** would it feel appropriate?

Peter Mac
Peter MacCallum Cancer Centre
Victoria Australia

05

NEXT STEPS

Summarise each groups responses and summaries – send out for comments

Member checking of the final maps: Send final process map for comments

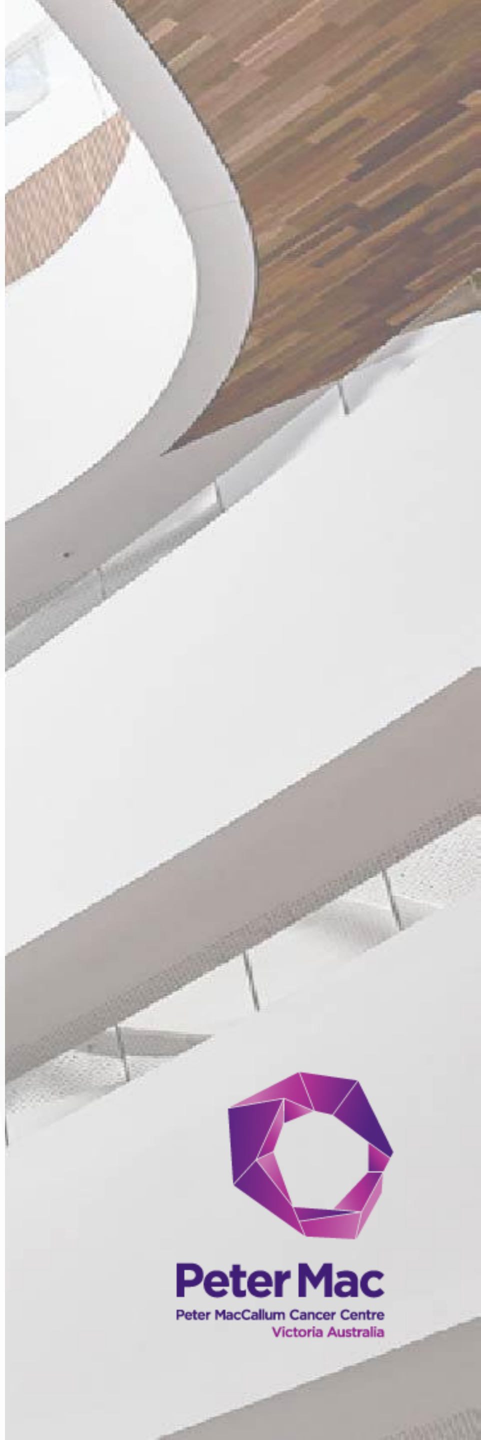

Peter Mac
Peter MacCallum Cancer Centre
Victoria Australia

Thank you

Peter Mac
Peter MacCallum Cancer Centre
Victoria Australia
