# Supplementary material for: Mapping provider and consumer voices using the AACTT framework: a focus group study of advance care planning
Source: BMC Health Serv Res. 2025 Jan 21;25:115. doi: 10.1186/s12913-025-12240-8 (PMC11752742; doi:10.1186/s12913-025-12240-8)
Supplement: Supplementary file 2 — Supplementary Material 2. [file 12913_2025_12240_MOESM2_ESM.docx]

**Improving the uptake of advance care planning for people with cancer:**

**FOCUS GROUP 1 – HEALTHCARE PROFESSIONALS**

1. Welcome to everyone and a thankyou for agreeing to participate in this study and particularly for making the time to participate in today’s focus group.

**I would really like to start off with some introductions and in the interest of keeping as much time as possible reserved for our discussion would ask everyone to perhaps keep this brief.**

1. **INTRODUCTIONS**

I would like to start off today with a brief definition of ACP and an overview of what we know, what we have done so far and where we will be going within the scope of this project.

**Defining ACP**

These are some examples of how you may have experienced ACP – May be in the form of being asked questions like…. or it may be documenting the responses to some of these questions…or trying to access the answers to some of these already documented questions.

1. **STUDY SUMMARY**

**This project in essence is aiming to improve ACP.**

There are 3 phases to this study –

First phase is creating process maps – and we will do this through two focus groups and will identify the flow of opportunities for ACP from the perspectives of various stakeholders.

Phase two – we will be exploring the barriers and enablers of ACP.

Phase 3 – workshop exploring the feasibility of proposed strategies to improve ACP.

**TODAYS OBJECTIVES**So here today we have some of you who are doctors, nurses, allied health, and health admin and we are interested in capturing the involvement of different Actors in ACP (so who is doing what where). - so, to structure the discussion I’ll ask members to take turns in responding and then we will open up for comments from the whole group

By the end of today we would have created a process map – that visually describes the opportunities and touchpoints of ACP – for different actors in different settings.

1. **GROUND RULES – START RECORDING**

- Voluntary participation
- No right or wrong answers, only different points of view. Important to hear about a range of views from everyone here.
- What’s discussed during the session is not discussed outside of the focus group session.
- We are recording the session – feel free to switch off the video and change the name if you would feel more comfortable.
- Any information we use will be de-identified.
- My role as the moderator will be to guide the discussion but feel free to talk to each other.

**DO YOU CONSENT TO PARTICIPATE IN THIS STUDY? -**

1. **DISCUSSION – Creating a series of process maps.**

**First Discussion slide – Building off exiting literature and some of the work that has already been done in the ACP Improvement group we would like to present this figure which we as group will interact with and discuss.**

- The circles represent the phases of ACP that have been presented literature. We are interested in exploring ….
- What do these phases mean to you?
- Are there **other aspects of ACP** that you are involved in that sit outside the scope of these phases?
- ****Allocate additional actions across ACP phases******
- Within your role at petermac how are you involved with ACP – across which phases?
- Do your experiences with ACP fit into these phases or across multiple phases –
- Who are these phases relevant for?
- ****Allocate actors across actions******

**These are some of the context/settings in which ACP might occur across the hospital.**

- EXPLORE THESE SETTINGS WITH THE GROUP

Of course, we know that very few people follow one patient pathways – try to establish a general discussion about typically what could happen at each of these points.

**Thinking about your involvement in ACP where could this possibly occur?**

- Opportunities for each of the ACP phases across these settings – that are specific to your role.
- What happens in these settings – in a typical journey?
- Is there an optimal time for this to occur in this setting?
- When interactions occur in this setting where does that fit in your workflow?

**FOCUS GROUP 2 – CONSUMERS**

1. Welcome to everyone and a thank you for agreeing to participate in this study and particularly for making the time to participate in today’s focus group.

**I would really like to start off with some introductions and in the interest of keeping as much time as possible reserved for our discussion would ask everyone to perhaps keep this brief.**

1. **INTRODUCTIONS**

I would like to start off today with a brief definition of ACP and an overview of what we know, what we have done so far and where we will be going within the scope of this project.

**Defining ACP**

These are some examples of how you may have experienced ACP – May be in the form of being asked questions like…. or it may be documenting the responses to some of these questions…or trying to access the answers to some of these already documented questions.

1. **STUDY SUMMARY**

**This project in essence is aiming to improve ACP.**

There are 3 phases to this study –

First phase is creating process maps – and we will do this through two focus groups and will identify the flow of opportunities for ACP from the perspectives of various stakeholders.

Phase two – we will be exploring the barriers and enablers of ACP.

Phase 3 – workshop exploring the feasibility of proposed strategies to improve ACP.

**TODAYS OBJECTIVES**So here today with your expertise we would like to hear about your experiences.

- so, to structure the discussion I’ll asking some question and then we will open up the discussion to the whole group

Today we will create a process map – that visually describe the opportunities and touchpoints of ACP – things like when is it appropriate to have these conversations? Who is the best person to have these conversations with? Where it is most appropriate for this conversation to happen?

**GROUD RULES – START RECORDING**

- Voluntary participation
- No right or wrong answers, only different points of view. Important to hear about a range of views from everyone here.
- What’s discussed during the session is not discussed outside of the focus group session.
- We are recording the session – feel free to switch off the video and change the name if you would feel more comfortable.
- Any information we use will be de-identified.
- My role as the moderator will be to guide the discussion but feel free to talk to each other.

**DO YOU CONSENT TO PARTICIPATE IN THIS STUDY? -**

1. **DISCUSSION – Creating a series of process maps.**

**Building off exiting literature and some of the work that has already been done in the ACP Improvement group we would like to present this figure which we as group will interact with.**

**These are phases of ACP that have been proposed in the literature – each of you may have experiences with some or all these phases in different ways and we are really interested in hearing about that.**

**The arrows on this figure represent the fact that this process is not always linear - and don’t always necessarily happen.**

1. Thinking about your own experience – what did the process of ACP look like for you? What might be missing?

**Allocate additional actions across ACP phases****

1. Through you own experiences when would it feel appropriate to start engaging with any of these phases of ACP?

Prompts: when is the right time for someone who has cancer to start preparing for or having the conversation? When is it appropriate?

1. Through your own experiences who are the most appropriate people to be involved? And in what setting?

**Allocate actors across actions****
